# The dynamical formation of ephemeral groups on networks and their effects on epidemics spreading

**DOI:** 10.1038/s41598-021-04589-7

**Published:** 2022-01-13

**Authors:** Marco Cremonini, Samira Maghool

**Affiliations:** 1 Department of Political and Social Sciences, University of Milan, Milan, Italy; 2 Department of Computer Science, University of Milan, Milan, Italy

**Keywords:** Computational science, Coevolution, Social evolution, Cultural evolution, Human behaviour

## Abstract

In network models of propagation processes, the individual, microscopic level perspective is the norm, with aggregations studied as possible outcomes. On the contrary, we adopted a mesoscale perspective with groups as the core element and in this sense we present a novel agent-group dynamic model of propagation in networks. In particular, we focus on ephemeral groups that dynamically form, create new links, and dissolve. The experiments simulated 160 model configurations and produced results describing cases of consecutive and non-consecutive dynamic grouping, bounded or unbounded in the number of repetitions. Results revealed the existence of complex dynamics and multiple behaviors. An efficiency metric is introduced to compare the different cases. A Null Model analysis disclosed a pattern in the difference between the group and random models, varying with the size of groups. Our findings indicate that a mesoscopic construct like the ephemeral group, based on assumptions about social behavior and absent any microscopic level change, could produce and describe complex propagation dynamics. A conclusion is that agent-group dynamic models may represent a powerful approach for modelers and a promising new direction for future research in models of coevolution between propagation and behavior in society.

## Introduction

In this work, we study the effects of the spontaneous formation of ephemeral groups during the declining part of a SIR-type propagation process^1^ and the possibility that those temporary groups could ignite a new propagation dynamic. The model takes inspiration from what we learned during the COVID-19 pandemic: increased urban mobility and gatherings have been observed not just as a consequence of the decision of health authorities to remove social restrictions; instead the phenomena has been documented starting right after the peak of the infection and increasing while approaching the lifting of social restrictions^2^. This spontaneous behavior, seemingly anticipating the decisions of health authorities, has effects that make the epidemic dynamics in the declining part a co-evolutionary process with a rich behavioral component still largely unaccounted in research. Most likely reasons for ephemeral groups formation could be the increasing psychological fatigue in complying with rules of social isolation following a period of compulsory seclusion and deprivation of social gatherings and activities^3–5^, distrust in health authorities and decay in risk awareness^6^, socio-economic disparities^7^, or seasonality effects like amenable weather conditions favoring gatherings in public places. It is probably safe to assume that a combination of several factors is at play in what appears in many cases a recurrent pattern: generalized strict social distancing has proved to be unattainable during the declining epidemic phase, even in presence of unsafe contagion rates. More generally, a large body of scientific literature exists on collective behaviors that epidemic models still do not fully address^8,9^ and the case of the COVID-19 pandemic makes no exception^10,11^.

Several recent COVID-19 works have shifted their focus on the conditions characterizing the declining part of an epidemic process, when the rate of contagions sensibly drops and social restriction measures are lifted, and on the combination of causes allowing for a relapse of the epidemic dynamics^12,13^. However, the vastness of the interplay between epidemic dynamics and behavioral responses remains still largely unexplored. Similarly, in the more general domain of co-evolutionary models of propagation and behavior, the study of dynamic network adaptations and the sheer variety of possible temporal phenomenon is still limited to few cases. This work moves in the direction of integrating an instance of collective behavior into agent-based network models of epidemics, with the specific focus on *ephemeral groups*, as we have dubbed the particular mesoscale structure of our interest. Two research goals have especially driven the work: first, to demonstrate the feasibility of our dynamic grouping technique in simulating network effects able to capture the behaviors produced by ephemeral groups of different characteristics. Secondly, to study and compare the effects of large and small ephemeral groups on the dynamics. In particular, we compare effects produced by scenarios with many small groups, which with the decreasing of their size are less likely to include an infected agent, with scenarios with few large groups.

Results show that a rich variety of dynamics could be reproduced with the combination of two network components, one static representing the traditional aggregated propagation based on slow social network changes and one dynamic representing the dynamic formation of ephemeral groups. Results also suggest that effects of a large group could be equally produced by simultaneous small groups, even in presence of a high rate of non-spreading groups. In the following sections, we first introduce and motivate the notion of ephemeral groups, then we describe the methodology and relevant simulation results for the different model configurations, each representing a stylized case study. We conclude discussing our findings and with some final remarks. This manuscript is completed by Supplementary Information presenting details about network characteristics, model states, and algorithms. In addition and of particular relevance is the *Null Model analysis* evaluating our ephemeral groups model with respect to two random models and a special case considering a non-overlapping version of our model.

## Ephemeral groups formation

The adoption of dynamic networks in epidemic models has frequently focused the attention of researchers on two main aspects: first, how network metrics developed for static networks should be modified when a dynamic network is considered^14^; second, the characteristic burstiness of human contact networks, which has been thoroughly investigated in different domains, from human mobility and communication, to physical contacts as the contagion vector^15–18^. Group formation, instead, has been extensively studied in network science, mostly in the form of generative models and community detection algorithms^19–21^. However, with respect to the study of microscopic structural features (agent specific), mesoscopic structural features (group specific) influencing a propagation dynamic have generally received less attention^22–24^. However, some notable works with specific focus on mesoscopic features have recently appeared: in COVID-19 research^25^, for the most general case of high-order interactions in complex systems^26^, and regarding high-resolution models of human mixing patterns and mobility, which define contact patterns and adopt mesoscopic descriptions^27,28^. High-resolution models, in particular, being data-driven, could be the ideal complement of a theoretical work like ours, providing the much needed case studies and real examples of mesoscopic structures necessary for realistic simulations and further model refinements and extensions.

In our model, *ephemeral groups* are mesoscopic structural components defined as spontaneous, temporary, and unstructured. They are mostly composed by unrelated individuals casually gathering together with no central planning, coordination, or organizational rules. They are typically small-to-medium sized and may represent, for instance, occasional parties or other unplanned assemblage in public or private spaces aimed at gregarious activities or random people finding themselves in close proximity, for a limited time span, near points-of-interests, in public transportation, or commercial locations. Ephemeral groups share the properties of possibly form simultaneously, repeatedly, and temporarily mixing individuals, often at random. Different is for large gatherings, which are likely to happen for special social events or circumstances, like conventions, sport competitions, fairs, or seasonal touristic influx in traditional locations. Usually, they are unique events, at least in a certain place and within a time period. Several cases of large social events have been studied with respect to their possible impact on contagions as superspreader events^29–31^. Ephemeral groups, individually, are not superspreader events, whereas it is possible for their combined effect. In addition, they are more difficult to study empirically than single large events. The relatively few exceptions have mostly regarded the case of people in public transportation, with data collected from turnstiles^32–35^, and scientific experiments involving limited groups of participants equipped with tracking devices (e.g., students on campus, visitors of museum exhibitions)^36,37^. We are interested to study the possible effects that those ephemeral groups may have on a possible new surge in a propagation process. The model has two components, which co-evolve producing mutually dependent network effects on the dynamic:

- *Microscopic static features* An extension of the traditional Susceptible-Infected-Recovered (*SIR*) model based on a static proximity network and simple contagion mechanism. This part has been originally presented in Ref.^38^ to study the epidemic dynamics under the assumption of different rates of Mild/Acute infected. It provides the base epidemic model and static contact network. Details about the basic epidemic model and model epidemic states are provided in Supplementary Information, Section S1 and S2.
- *Mesoscopic dynamic features* The dynamic formation of ephemeral groups is modeled as a choice of randomly selected agents that, starting from a given time point in the declining epidemic phase (more on the criteria for selecting when to start ephemeral groups in Supplementary Information, Section S2, subsection *Ephemeral groups creation pseudocode*), dynamically link pairwise in groups having the following characteristics: a variable rate of agents is involved in groups; groups are randomly created and possibly repeated for more time steps, each group may or may not include infectious agents (i.e., the probability to include spreading agents is increasing with the group size).

**Figure 1:**
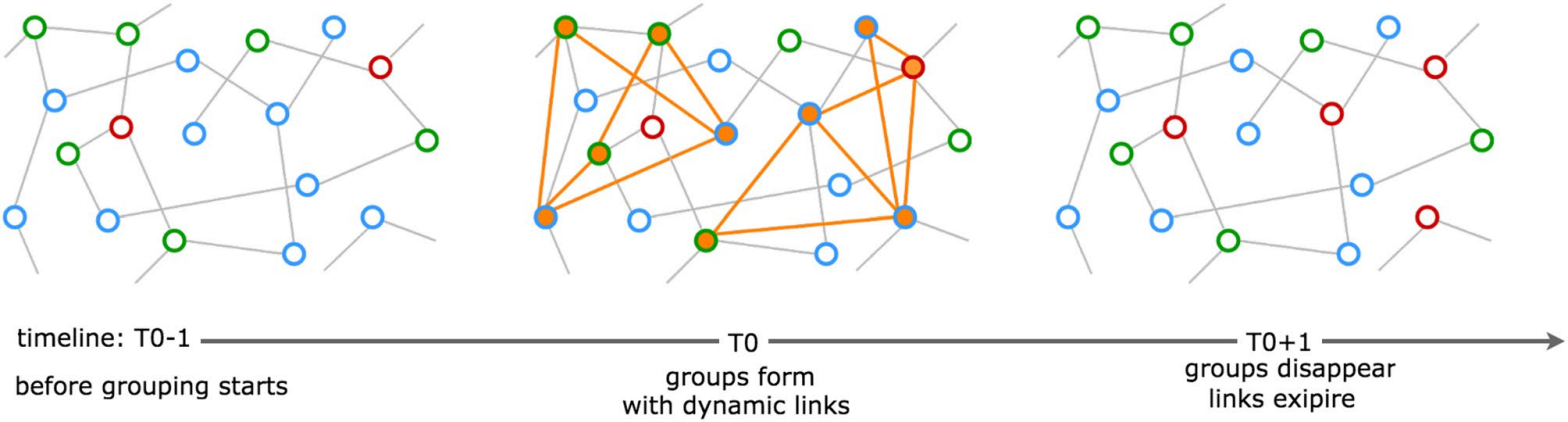
The contact network is described through static links and agent states. Red is for Infected (e.g., spreading agents, infected individuals in disease epidemics, adopters in product adoption, spreaders in opinion diffusion), green is for Recovered (e.g., agents no longer spreading, immunized, stifler, contrarian, antagonist, deluded are possible definitions in different contexts), and blue is for Susceptible (e.g., agents possibly becoming spreading actors, ignorant, undecided, laggard); filled in orange are agents belonging to an ephemeral group.

Figure 1 shows a schematic representation of ephemeral groups effects studied in this work.

On the timeline of simulation steps (time steps), the network dynamic is described with the progression of three characteristic steps:

- *Before ephemeral groups formation* (*time step* $T0-1$) The system configuration is the product of the static contact network evolution and agents’ state transitions. Specifically, by focusing on the declining part of the dynamic, we consider the system as slowly evolving, with few agents still changing state. In the example of Fig. 1, the two red agents (Infected, for simplicity) are no longer spreading because connected only to green agents (Recovered). In the bottom right corner, a blue agent (Susceptible) appears as disconnected from the visible portion of network.
- *During ephemeral groups dynamic linking* (*time step* *T*0) Two groups of five agents with two new links for each agent are showed marked in orange in the central vignette. Node selection for each group is random, in this version of the model. Dynamic links are assumed to be intragroup and randomized as well. Propagation from red (I) to blue (S) agents is, therefore, a first order effect of grouping only between agents belonging to the same group. Propagation to external agents is a second order effect. For simplicity, no social learning or herding effects on propagation have been assumed. Ephemeral groups are assumed to have a typical single time step lifetime. Different configurations based on the repetition of the dynamic grouping for more time steps have been simulated and studied.
- *After ephemeral groups dissolve and dynamic links expire* (*time step* $T0+1$): The creation of new links inside ephemeral groups logically reshuffles the topology of the contact network, leading to different effects based on the group composition. In the example of Fig. 1, one group has a red (I) agent among its members, therefore possibly spreading the phenomena, while the other has none, being inactive. Spreading could be enhanced by shortening paths, connecting agents previously disconnected, or offering new contacts to isolated spreading agents, as the third vignette shows with the red agent, previously isolated between two green ones, been able to turn red the disconnected bottom right blue agent and another blue agent, from which the propagation could start over on the static network.

## Materials and methods

### Model

We present a stochastic group-based network model focusing on the declining part of a schematic SIR-type propagation process in a society and considering the formation of ephemeral groups creating new dynamic links. The model is an extension and a generalization of a previously presented model^38^. It extends the previous model by studying the conditions of ephemeral groups leading to a second epidemic dynamic under different scenarios. It is a generalization because the analysis and conclusions have a broader aim than previous COVID-19 research, discussing cases of coevolution between propagation and behavior, when the behavioral component is expressed by a combination of individual (microscale model) and dynamic groups (mesoscale model). We have also simplified the analysis of agents’ state transition dynamics in order to better focus on the role of ephemeral groups from the previous extended SIR model (more details in Supplementary Information, Section S2). This way, combined with the assumption of fixed population, we have also restricted the effects produced by ephemeral groups only to a second propagation dynamic. Removing one of these assumptions and allowing for a renewal of the sub-population of agents that could turn into spreading ones (i.e., susceptible turning infected) would make possible to sustain a series of propagation dynamics through the formation of ephemeral groups. For this study, we adopt a constant population of agents $N=10,000$ connected on an abstract network with scale-free characteristics (Supplementary Information, Section S1 provides specific information about the network generation algorithm, the degree distribution, and some typical network centrality measures). The results of 160 different model configurations are presented, all referring to cases producing a recognizable response on the overall dynamic. They have been run through simulations and for each one we took a sample of 50 valid trials. A trial is considered *invalid* when the second wave was absent or insufficient to represent an epidemic dynamic (i.e., we assumed as the exclusion criteria to have less than 100 additional infected agents at the peak).

**Figure 2:**
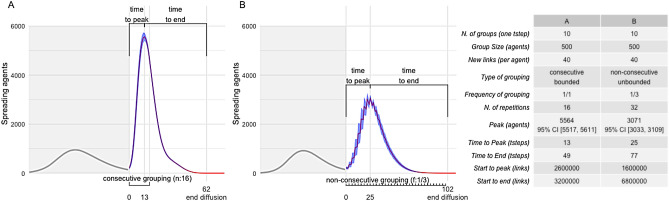
In panels (**A**) and (**B**), characteristic features used in reporting the results are showed. The *peak* of the process represents the maximum number of Infected. The peak time step is indicated on the x axis with the offset from the origin [+ 13 in panel (**A**) and + 25 in panel (**B**)]. *Time to peak* and *time to end* represent duration (in time steps). Asymmetry of the curve and dependence on different factors makes it useful to measure the two parts independently. *Start of grouping* and *end of diffusion* are typical points in the timeline, respectively, indicated with 0, corresponding to the flex of the curve and the first time step of dynamic grouping, and a point that varies for each configuration, by convention assumed as the time step when the majority of trials has no Infected agent left [indicated as an offset from the flex, + 62 in panel A and + 102 in panel (**B**)]. *Grouping pattern* and *duration* are indicated with horizontal brackets. For panel (**A**), grouping has been repeated for 16 consecutive time steps; for panel (**B**), grouping has a 1/3 frequency and run from start to end of diffusion. In both cases, we see that the peak of the dynamics, while depending on grouping, may occur before grouping ends. The table lists the parameters that describe each configuration. We will use the notation *#G* for the *number of groups* and ($y_1$=*group size*; $y_2$=*new links*; $y_3$=*grouping pattern*) to indicate a configuration, where the grouping pattern could be a finite number of steps *n* or a frequency *f*. The frequency could indicate consecutive grouping $f=1/1$ or periodic grouping $f=1/m$ (i.e., $f=1/3$ is the value we used in tests). For example: given **#G=10**, **(500; 40; 16)** identifies the configuration of panel (**A**), **(500; 40; 1/3)** identifies the configuration of panel (**B**).

Figure 2 shows two panels, A and B, representing the general classes of propagation dynamics studied in this work, and a table containing the characteristic parameters analyzed for all the configurations and presented in the results. The main difference between panel A and panel B lays in the pattern of grouping repetition: the *grouping frequency* (i.e., the number of grouping time steps over a certain interval) and the *total number of grouping time steps*. For the grouping frequency we have considered two cases: *continuous* when grouping occurs at consecutive time steps and *periodic* when grouping occurs at non-consecutive time steps. For the number of grouping time steps, we have considered two cases as well: *bounded* when a fixed number of grouping time steps is set and *unbounded* when grouping is allowed until the exhaustion of the propagation dynamics. In Fig. 2, panel A represents a continuous and bounded propagation dynamic, in this case 16 are the consecutive grouping time steps, indicated with $n=16$. Instead, panel B shows a periodic and unbounded propagation dynamic, with grouping occurring at a rate of one every three time steps, indicated as $f=1/3$, which produces the typical sawtooth shape of dynamic processes characterized by cyclic reinforcements followed by decline phases.

Panels A and B introduce several elements of the dynamics we have studied. The first part of the propagation dynamic, marked with the grey area, is useful only to have a general overview of the full process and of the typical proportions between the two phases of the propagation dynamics (several other cases with a second dynamics smaller than the first have been simulated, but in this work we focus specifically on those producing a large response). Other than that, the first part of the dynamic is not particularly relevant, representing equal initial conditions for ephemeral groups among all cases. Average number, with 95% CI, of Infected, Susceptible and Recovered agents at the start of ephemeral grouping are in Supplementary Table S2 of Supplementary Information. In following figures, the first epidemic wave will be omitted. A detail to highlight is that we studied the second propagation dynamic as the result of a continuous propagation process that produces a first dynamic, then, when a threshold on the number of spreading agents (I) is reached in the declining phase, it triggers the dynamic grouping mechanism and a flex is produced, starting the second dynamic. To be more specific, for each configuration, we have aligned all trials with respect to the flex point in order to normalize the time-dependent variability and then averaged over the trials. The first dynamic (i.e., the grayed area up to the flex of panels A and B ) is independent from the grouping mechanism and shared by all configurations, with a variability within narrow stochastic bounds.

The cases we analyzed have been categorized in scenarios, each defined by the number of ephemeral groups produced per time step ($\#G$), and for each scenario in configurations. Together, group size, number of new links per agent, the already introduced grouping pattern, and the number of groups uniquely identify each configuration presented in the results. More specifically,

- *Group size* represents the number of agents belonging to a group. In this study, we assume that every configuration has groups of equal size. When grouping is repeated for more time steps, at each time step groups are recreated randomly. Overlaps between groups are possible and in general high-order coupling could have relevant effects on the dynamics. For this reason, we tested a non-overlapping variant. The model analysis of statistical significance is presented in Supplementary Information, Section S4, showing that, in our case, group overlaps seems to have a negligible effect.
- *New links per agent* represents the number of new links each agent randomly creates. Dynamic links are only intragroup and have a lifetime of one time step. When grouping is repeated, continuously or periodically, dynamic links are recreated every grouping time step. In this study, we assume same number of new links per agent for each configuration. Dynamic links are removed when a group dissolves.
- *Number of groups* represents how diffuse the ephemeral grouping behavior is assumed to be in the population. As a rule of thumb, we assume an inverse proportion between number of groups and their size, that is, we investigated cases of many small groups and of few large groups in order to compare the behavior. The total number of agents involved in ephemeral groups is then calculated as *N. of groups* $\cdot$ *group size*, while the total number of dynamic links for each grouping time step is *N. of groups* $\cdot$ *group size* $\cdot$ *new links per agent*.

Pseudocode describing the algorithms could be found in Supplementary Information, Section S2.

## Results

Model configurations have been divided in four scenarios, each one assuming a different number of groups per time step, $\#G =(1,10,100,1000)$. The choice of these values has been driven by the extremes: on the one side we wish to test the case of a single large group ($\#G=1$), which mimics a large gathering of loosely connected agents, and on the other the case of a multitude of small groups ($\#G=1000$), tightly connected by dynamic links but in large majority inactive because without spreading agents. The other two values ($\#G=(10,100)$) represent intermediate scenarios. For each scenario, eight grouping patterns have been considered, six bounded with number of time steps $n=(1,2,4,8,12,16)$ and two unbounded with frequency $f=(1/3, 1/1)$.

Base epidemic and ephemeral groups simulation settings are listed in Supplementary Table S2 of Supplementary Information. For convenience, we report here the initial conditions of agent states when ephemeral groups start. Values are calculated from the trials taken from a sample of configurations and are in the form *average [CI 95%]*: *Infected*: 140 [142.25, 137.75]; *Susceptible*: 7087 [7122.24, 7051.76]; *Recovered*: 2773 [2806.25, 2739.85]. For each scenario, eight grouping patterns have been considered, six bounded with number of time steps $n=(1,2,4,8,12,16)$ and two unbounded with frequency $f=(1/3, 1/1)$.

### Consecutive grouping, bounded and unbounded

**Figure 3:**
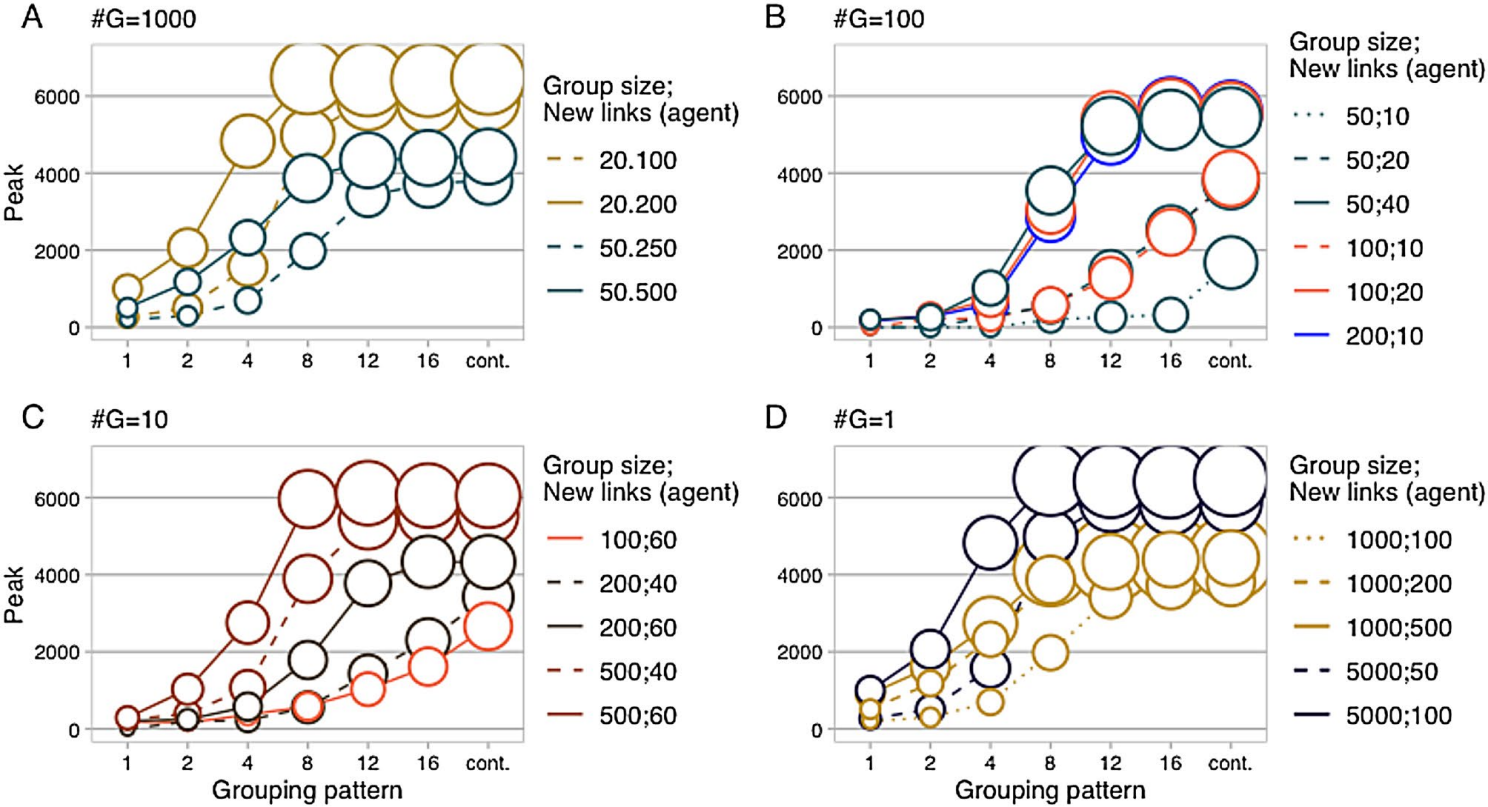
Six model parameters are represented for a total of 140 configurations run through multi-agent simulations (the 20 cases with $f=1/3$ are not included here). For each configuration, a sample of 50 trials has been took, aligned with respect to the flex, and averaged. Each circle represents a single configuration [28 in panel (**A**), 42 in panel (**B**), 35 in panel (**C**) and (**D**)]. Parameter description with the corresponding graphic element follows. Panel: number of groups created per time step; x axis: grouping pattern for six consecutive and bounded cases $n=(1,2,4,8,12,16)$ plus the case consecutive and unbounded $f=1/1$; y axis: peak size as number of spreading agents; color and linetype: group size and new links per agent; area of circles: total new links ($\times$ 1000) created by all groups from start of grouping (flex) to the peak (new links possibly created after the peak are not part of the metric). From panels (**A**–**D**), it derives that configurations that plateaued reached the stationary peak level within 8–12 consecutive grouping repetitions, while grouping patterns of 16 or unlimited time steps are mostly relevant for configurations that produced a weaker response.

We first look at results from configurations with consecutive grouping time steps, bounded in the number of repetitions of group formation with $n=(1,2,4,8,12,16)$ and unbounded (frequency $f=1/1$). Configurations with non-consecutive grouping (frequency $f=1/3$) have peculiar characteristics we discuss later. Results are first presented in Fig. 3 to give a broad overview of the outcome from all configurations and to present observations based on peak values mostly. Then, a more detailed analysis focusing on a subset of configurations and introducing productivity based and time based metrics is summarized in Table 1.

**Table 1:** Configurations are categorized by number of groups $\#G$ in the four quadrants, then by series (*group size*, *new links per agent*) in columns, and finally by grouping patterns, here only $n=(8,12,16)$ and $f=1/1$, in rows. The values of the first four rows are those of the $K_{Eff} (\times 1000)$ efficiency metric, with average efficiency of the ephemeral grouping process in the fifth row. The black triangle ($\blacktriangle$) corresponds to the grouping pattern that reached first the peak for each configuration. The last four rows of each quadrant are referred to the pattern with the black triangle $\blacktriangle$ and show: the peak value, the two time based metrics *time to peak* and *time to end*, and* links to peak* ($\times$ 1000), representing the number of links ($\times$ 1000) produced by groups from start of grouping to peak. For example, first model configuration of the first quadrant (50:10 of #G=1000): the $\blacktriangle$ is on the row corresponding to grouping pattern $n=12$, therefore values of the last four lines should be meant as referred to that pattern.

N. of groups #G=1000					N. of groups #G=100						
	50; 10	50; 5	20; 10	20; 5		200; 10	100; 20	50; 40	100; 10	50; 20	50; 10
8	1.62	1.91	1.10	0.37	8	1.68	1.82	2.12	0.55	0.52	0.15
12	1.67 $\blacktriangle$	1.95 $\blacktriangle$	1.96	0.63	12	2.02	2.18	2.12	0.95	1.09	0.23
16	1.70	1.77	1.71	1.24	16	1.84 $\blacktriangle$	1.96 $\blacktriangle$	2.02 $\blacktriangle$	1.55	1.50	0.28
1/1	1.68	1.81	1.76 $\blacktriangle$	1.39 $\blacktriangle$	1/1	1.94	1.93	2.04	1.68 $\blacktriangle$	1.65 $\blacktriangle$	0.78 $\blacktriangle$
Avg.	1.67	1.86	1.63	0.91	Avg.	1.87	1.97	2.07	1.18	1.19	0.36
Peak $\blacktriangle$	6816	5999	5284	3604	Peak $\blacktriangle$	5652	5635	5444	3861	3783	1678
Time to peak	8	13	15	26	Time to peak	14	14	13	23	22	40
Time to end	46	47	50	69	Time to end	47	49	52	62	65	92
Links to peak ($\times$ 1000)	4000	3250	3000	2500	Links to peak ($\times$ 1000)	2800	2800	2600	2300	2200	1950

N. of groups #G=10						N. of groups #G=1					
	500; 60	500; 40	200; 60	200; 40	100; 60		5000; 100	5000; 50	1000; 500	1000; 200	1000; 100
8	2.42 $\blacktriangle$	2.34	1.73	0.67	0.94	8	1.59 $\blacktriangle$	2.42	1.00	2.32	2.30
12	2.00	2.20 $\blacktriangle$	2.52	1.34	1.24	12	1.57	2.10 $\blacktriangle$	0.78 $\blacktriangle$	1.93 $\blacktriangle$	2.72
16	1.97	2.09	2.18 $\blacktriangle$	1.69	1.55	16	1.57	2.07	0.78	1.93	2.25 $\blacktriangle$
1/1	1.97	2.08	2.05	1.86 $\blacktriangle$	1.62 $\blacktriangle$	1/1	1.58	2.09	0.78	1.95	2.30
Avg.	2.09	2.18	2.12	1.39	1.34	Avg.	1.58	2.17	0.84	2.03	2.39
Peak $\blacktriangle$	6115	5564	4334	3424	2662	Peak $\blacktriangle$	6483	5887	4416	4392	3807
Time to peak	10	13	16	22	26	Time to peak	8	11	11	11	16
Time to end	49	49	54	73	73	Time to end	48	48	53	53	56
Links to peak ($\times$ 1000)	3000	2600	1920	1760	1560	Links to peak ($\times$ 1000)	4000	2750	5500	2200	1600

#### Peak analysis

By considering peak values of Infected agents reached by the different cases, we compare the performances in different scenarios, grouping patterns, and number of dynamic links created up to the peak value.

Very large peak values, in our experiments close or above 6000, mean that the propagation process reached the maximum extension and was capped by the population size and the proportion of agents that cannot be turned into spreading ones (Recovered). Configurations in all scenarios have easily reached such strong responses in a range of parameter values (group size, number of new links per agent, and number of grouping time steps), either with few loosely connected groups (e.g., lesser than 1/10 for the ratio of new links per agent over the group size) or with many small tightly connected groups (e.g., ratio of new links per agent over the group size greater than 1/10).

Therefore, from Fig. 3, a first observation is that a strong propagation dynamic cannot be excluded or it cannot be considered more or less likely only by observing whether the scenario is composed by few large but loosely connected groups (as for panels C and D) or many small but tightly connected groups (as for panels A and B).

Secondly, a plateau is not been reached only as a consequence of bounded network size, but it could be the result of a spontaneous behavior of the propagation process. This is showed in panels C (see configuration 200; 60) and particularly in panel D (see the whole series 1000; 100, 1000; 200, and 1000; 500), where peaks reach a medium level around 4000, far from the dominant effect of the limited network size (at that level, the rate of Recovered and Susceptible agents is around 30%). More specifically, in panel D, the series with group size equals to 1000 shows that the peak stays around 4000 for a large range of new links per agent (from 100 to 500). It also exhibits a striking correspondence with the behavior of the series with 5000 as group size reaching the peak value with 8 consecutive grouping time steps and similar number of total new links produced by the group, then plateaued. Other cases in different scenarios behave similarly (e,g, the 50; 10 of panel A or the 100; 60 of panel C). Another set of cases (i.e., 20; 5 of panel A; 100; 10, 50; 20 and 50; 10 of panel B; 200; 40 and 100; 60 of panel C) have a slower growth and reach the peak value only when we tested with an unbounded number of grouping time steps (for those cases, more than 20 and up to 40 consecutive grouping time steps were necessary to reach the peak).

A positive correlation between the peak level and the total number of links created by groups up to the peak ($\#G \cdot group\; size\; \cdot new\; links\; per\; agent \cdot time\; to\; peak$) could be observed, but the relation is not linear and could be capped. Series presented in panel B of Fig. 3 have an almost perfect correlation for all grouping patterns (i.e., 50; 40, 100; 20, and 200; 10 overlaps, as well as 100; 10 and 50; 20). In panel A and C, the correlation is still clear but, for each configuration, only up to the grouping pattern that plateaued. To this regard, panel D is the most interesting, with the capped peak of the 1000 series. Configuration 200, 40 of panel C provides another example of series that plateaued at mid-range of peak values.

#### Efficiency of dynamic linking

*Efficiency* is a typical property of a process measured as the ratio between the output of the process to the input, representing which fraction of output could be obtained by consuming a unit of input. Expressed as costs or assets for the input, and number of items produced by the process for the output, an *efficiency metric* is useful for comparing processes and rank them based on how efficiently they use the resources, monetary or operation. Said differently, an efficiency metrics measures the *marginal gain of resources.*

In our model, we have observed that the peak of the epidemic propagation depends on the number of dynamic links up to the peak time step. Therefore, a measure of efficiency of ephemeral grouping processes could be done by considering the total number of Infected agents created during the time to peak phase as the output, and the total number of dynamic links created during the time to peak phase as the input. The model configuration that needs fewer dynamic links to achieve a new Infected agent is the most efficient. Eq. 1 shows the formula.

1 $$\begin{aligned} K_{Eff}=\frac{\sum Infected(t_{peak})-\sum Infected(t_0)}{\sum _{i=t_0}^{t_{peak}}dynLinks(i)} \qquad with\ dynLinks(i) = \#G \cdot group\; size \cdot new\; links\; per\; agent. \end{aligned}$$

More precisely, the $K_{Efficiency}$ metric is an approximation of the grouping process efficiency, because it does not distinguish between new Infected agents directly produced by dynamic links and those instead created by the epidemic dynamics on the static contact network. However, considering that it is only thanks to the ephemeral groups mechanism that the epidemic has produced a second wave, in first approximation we assume that all new Infected agents are, directly or indirectly, produced by the grouping process.

Table 1 presents the values of $K_{Eff} (\times 1000)$ (in the first five rows of each quadrant), so that different model settings (pattern, configurations, scenarios) could be compared in terms of efficiency of the conversion of dynamic links into Infected agents. The last four rows of each quadrant give information related to the pattern first reaching the peak (peak value, time to peak, time to end, and links to peak).

Values $K_{Eff}(\times 1000)<1$ represent cases of slow and ineffective propagation, whereas values $K_{Eff}(\times 1000)>2$ are associated to fast and efficient cases. Next, the positive correlation between the peak value and the number of dynamic links created in the time to peak interval is confirmed for all series with one exception: (1000; 500). As a matter of fact, the whole (1000; *) series is a special case, because it shows how the marginal gain in Infected at the peak could decrease with the increasing density of dynamic links: (1000; 100) produces the lowest peak but with highest $K_{Eff}$, then the peak increases for (1000; 200) but with a reduced marginal gain, until the (1000; 500) case that shows a small marginal gain and a clearly inefficient propagation, up to the point of losing the positive correlation showed by the other cases.

Another correlation that is observed is that *time to peak* and *time to end* are negatively correlated with the peak value and the* links to peak* ($\times$* 1000*). This is not a new result in general, on the contrary, it is typical of SIR-type dynamics^1,39^. What is possibly interesting is the fact that the relation between peak value, number of links and duration of the propagation process could be controlled, to some extent, by the grouping mechanism. This offers interesting possibilities, that we further investigate in the next section.

### Non-consecutive grouping

Table 2 shows some results from a slightly different set of experiments aimed at investigating the effects of periodic ephemeral groups that activate with a non-consecutive pattern. What we present is our first take, initial results, and analysis of an unexplored group model of propagation dynamics and behavior, to the best of our knowledge, which, we guess, could have numerous interesting variants and applications to many areas. Non-consecutive grouping obviously reduces the total number of dynamic links created in a certain time span with respect to the consecutive case, but we know that the peak is correlated with the number of links created up to the peak, so a first result was to compare the peak analyses in the two cases, consecutive and non-consecutive. Secondly, periodic grouping may produce, as we have showed in Fig. 2 for $f=1/3$, the typical sawtooth shape of a function that alternates fast growth with a slower decline due to the fast rise of new spreading agents produced by groups followed by the slower dynamic on the static contact network where the rate of conversion from spreading agents (I) to inactive agents (R) is higher than those from potentially spreading (S) to spreading. Therefore, an interesting question regards the possibility to prolong or even fully sustain a propagation dynamic by means of a proper tuning of periodic grouping. Table 2 offers some insights. Comparing peaks analyses:

- with $f=1/3$, for each group size, the peak value and the number of links up to the peak are still positively correlated, but the correlation is lost among series of different group size;
- considering the difference with consecutive cases, peaks for $f=1/3$ fall faster than the link numbers, with the exception of cases with $\#G=1$; this implies a reduced marginal efficacy of new links, confirmed by negative differences of the $K_{Eff}$ index;
- in $\#G=1$, $K_{Eff}$ differences are all positive, except for (1000; 100) and peaks do not fall faster than link numbers.

Instead, with respect to the ability of a non-consecutive grouping pattern to extend the duration of the propagation, results show *time to peak* having increments from 50 to more than 100%. This was expected because it mostly depends from the reduced total number of links from start to peak. The more interesting result is that *time to end* has large increments, from 30 to more than 100%. Notable is that those are increments with respect to the consecutive case $f=1/1$ that repeats grouping until the end of propagation and creates three times the number of links of $f=1/3$. Again an important case where more links do not produce more propagation, in fact after the peak $f=1/1$ has a negative marginal gain with respect to $f=1/3$.

**Table 2:** Table layout is similar to Table 1. For each quadrant, the values of the first row are those of the $K_{eff} (\times 1000)$ index for the non-consecutive grouping pattern with $f=1/3$. The following *diff %* row, show the percent difference with the corresponding values of average efficiency, (*avg.*) row of Table 1. All other *diff %* rows refers to corresponding rows of Table 1.

N. of groups #G=1000					N. of groups #G=100						
	50; 10	50; 5	20; 10	20; 5		200; 10	100; 20	50; 40	100; 10	50; 20	^a^50; 10
1/3	1.98	1.84	1.39	0.53	1/3	1.15	1.83	2.07	0.84	0.75	0.14
diff %	18.82	− 1.05	− 14.87	− 41.55	diff %	− 38.53	− 7.22	− 0.19	− 28.93	− 37.01	− 61.05
Peak	5087	3232	2494	694	Peak	2733	2845	2883	820	918	125
diff %	− 25.37	− 46.12	− 52.80	− 80.74	diff %	− 51.65	− 49.51	− 47.04	− 78.76	− 75.73	− 92.55
Time to peak	13	28	30	51	Time to peak	31	28	25	52	48	1
diff %	62.50	115.38	100.00	96.15	diff %	121.43	100.00	92.31	126.09	118.18	− 97.50
Time to end	59	71	88	136	Time to end	77	79	80	113	115	202
diff %	28.26	51.06	76.00	97.10	diff %	63.83	61.22	53.85	82.26	76.92	119.57
Links to peak ($\times$ 1000)	2500	2250	2000	1700	Links to peak ($\times$ 1000)	2200	1800	1600	1500	1600	50
diff %	− 37.50	− 30.77	− 33.33	− 32.00	diff %	− 21.43	− 35.71	− 38.46	− 34.78	− 27.27	− 97.44

N. of groups #G=10						N. of groups #G=1					
	500; 60	500; 40	200; 60	200; 40	100; 60		5000; 100	5000; 50	1000; 500	1000; 200	1000; 100
1/3	2.38	2.10	1.83	0.98	0.94	1/3	2.09	2.26	1.15	2.13	2.08
diff %	13.80	− 3.61	− 13.72	− 29.61	− 29.77	diff %	32.49	4.15	37.72	4.80	− 13.06
Peak	4074	3071	1931	848	651	Peak	4918	3708	2743	2697	1735
diff %	− 33.38	− 44.81	− 55.45	− 75.23	− 75.54	diff %	− 24.14	− 37.01	− 37.88	− 38.59	− 54.43
Time to peak	16	25	31	45	52	Time to peak	13	18	13	16	28
diff %	60.00	92.31	93.75	104.55	100.00	diff %	62.50	63.64	18.18	45.45	75.00
Time to end	72	77	93	117	131	Time to end	63	76	113	117	110
diff %	46.94	57.14	72.22	60.27	79.45	diff %	31.25	58.33	113.21	120.75	96.43
Links to peak ($\times$ 1000)	1800	1600	1320	1280	1020	Links to peak ($\times$ 1000)	2500	1750	2500	1200	1000
diff %	− 40.00	− 38.46	− 31.25	− 27.27	− 34.62	diff %	− 37.50	− 36.36	− 54.55	− 45.45	− 37.50

^a^Series 50, 10 for f = 1/3 produces a negligible second dynamic.

## Discussion

In this work, we have proposed a new dynamic group-based network model of a propagation process, specifically focusing on modeling mesoscopic constructs. Ephemeral groups are the composite subjects of our model. Model simulations, analysis of results and Null Model analysis (presented in Supplementary Information, Section S3) permit us to draw some conclusions.

First, by considering where we started from, an epidemic model inspired by COVID-19 pandemic, we observe that ephemeral groups could be a relevant factor in epidemic dynamics when people return to socialize freely. Large gatherings resulting in superspreading events have been frequently discussed, as well as repetitive gatherings protracted for long periods, such as in schools or workplaces. Ephemeral groups are instead more elusive to a rigorous assessment of the risk they pose. To this regard, future models may shed new light on the relevance of groups on epidemic dynamics. An outcome of our work is that, at least in theory, for several and diverse configurations, ephemeral groups have the power to restart and sustain an epidemic dynamic when it was headed to expiration, in the absence of contextual changes (e.g., new more transmissible pathogens). Health authorities should exercise prudence in face of the inevitable uncertainty regarding the effects of gatherings and the difficulty of scientists in collecting precise measurements to feed epidemic models.

Secondly, the *time to end* metric seemingly confirms the hypothesis that a periodic dynamic grouping mechanism could deeply influence the propagation process and could sustain it for an extended period. This result could remind of known behavioral and cognitive effects from social learning theory^40^ or awareness models^41^, all implying that periodic reinforcements could sustain an otherwise decaying process. A novelty of our work is to have derived some initial quantitative results through simulations of a group model, with the analysis suggesting that balancing the alternation of growth and decay phases, rather than simply the number of reinforcement events, is a key factor and it would be the basis for new and possibly interesting future research in this area.

Another outcome that looks interesting comes from the Null Model analysis. It suggests a change of the relevance of groups through the different scenarios. Many small groups, most of them with no internal epidemic propagation because without infected members, seem to act similarly to a random linking model. But they could be nevertheless effective in sustaining an epidemic dynamic. In real settings, they could possibly join to form larger groups. In larger groups the difference with random linking models emerges increasingly clear. Simulation data show that the ephemeral groups model reaches often a slightly higher peak with fewer active links (i.e, links between an Infected and a Susceptible agent) than corresponding random cases. This seems to indicate that groups seed and sustain a more efficient epidemic propagation. A dynamic of groups aggregating to form larger compounds represents a behavioral phenomena potentially at the base of particularly meaningful propagation models. On the practical side, not just the creation of ephemeral groups and their characteristics are relevant in a propagation phenomena, but the temporal evolution of groups organization is important as well.

Other observations are probably less relevant, but still worth mentioning. From Table 1, we obtained a detailed description of the dynamics produced by the different cases of consecutive grouping. The total number of links created up to the peak is the factor that determines the peak level (positively correlated) and the time needed to reach it (negatively correlated), which implies the existence of feedback loops in the dynamic. An important limit to the correlation between peak and number of links is the case of a propagation process that plateaued, which happens when the ability of dynamic links to produce new spreading agents drops to values of the $K_{Eff} (\times 1000)<1$. That could happen due to the limited size of the population (i.e., the trivial case of saturation) or for contextual conditions of the propagation process for which, up from a certain level, increasing densities of dynamic links accentuate inefficiency in propagation and a decreasing marginal gain of links. The correlation is lost if the total number of links produced from the start of grouping to the end of propagation is considered, rather than only those up to the peak. Finally, non-consecutive grouping reveals interesting scenarios. The analysis of results from Table 2 disclosed a complex interplay between the two dynamics running in parallel, the one on the static contact network and the one of dynamic grouping. On the one side, the reduced marginal efficacy of dynamic links of the non-consecutive cases produces less new infected agents, and on the other it changes the balance between the two dynamics, shifting the weight in favor of the rate of agents recovering. This makes peaks falling, with a rate that accelerates with the loss of efficacy of dynamic links.

A concluding remark is that by studying this model, we had the clear feeling of how vast and rich could become models based on dynamic groups. Moving from typical epidemic models to models designed for the propagation of ideas, news, or products will keep some common features, but will introduce new set of assumptions, radically changing the dynamics. High-order coupling, here barely touched in Supplementary Information, group membership persistence, heterogeneous groups, time dependent agent behavior and social reinforcement, the influx and outflux of new agents, as well as the representation of different cultures in agent populations could all become key factors of future challenging as well as interesting models.

## Supplementary Information

Supplementary Information.

## Data Availability

Data are available at https://zenodo.org/record/5626804.
