# Supplementary material for: The dynamical formation of ephemeral groups on networks and their effects on epidemics spreading
Source: Sci Rep. 2022 Jan 13;12:683. doi: 10.1038/s41598-021-04589-7 (PMC8758734; doi:10.1038/s41598-021-04589-7)
Supplement: Supplementary file 1 — Supplementary Information. [file 41598_2021_4589_MOESM1_ESM.pdf]

Supplementary Information for:

The dynamical formation of ephemeral groups on networks and their effects on epidemics spreading

Marco Cremonini[1,*] **and Samira Maghool**[2,*]

[1]University of Milan, Department of Political and Social Sciences, Milan, Italy
[2]University of Milan, Department of Computer Science, Milan, Italy
[*]marco.cremonini@unimi.it
[*]samira.maghool@unimi.it

S1: Network information and metrics

The networks used by our models for experiments have a constant population of N=10000 and scale-free characteristics. They are generated through the `powerlaw_cluster_graph` function of the NetworkX package (https://networkx.github.io/documentation/networkx-1.10/reference/generated/networkx.generators.random_graphs.powerlaw_cluster_graph.html), which is based on Holme and Kim algorithm[1] for graphs with powerlaw degree distribution.

Figure S1 shows an example of degree distribution, while Table S1 presents some network metrics.

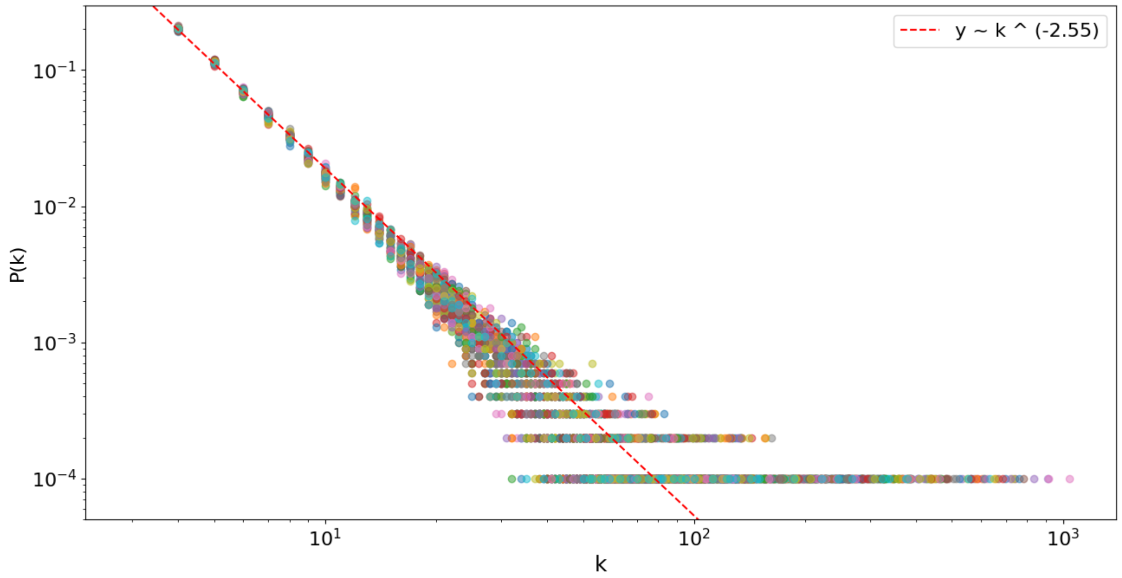

Figure S1. Degree distribution produced by trials used in simulating a single model configuration. Points of different colors correspond to different trials. The figure is on log-log scale, the red dashed line is the powerlaw fit. The distribution is typical of scale-free networks.

Table S1. Network centrality metrics.

Metric	Value
Average degree	5.99732
Average betweenness (normalized)	0.0003349
Average eigenvector	0.0035467
Average clustering	0.327954
Average Path length	4.3024

S2: Model states and execution

This section is divided in two subsections, respectively aimed at presenting:

- Model states, the state transition diagram, and pseudocode for model execution related to the epidemic spreading. The material of this subsection is not part of the original contribution of the present study, being taken from our previous work on a related subject[2]. In that first work, we have defined the original epidemic model and network that we have used in this work for the static contact network. This model produces the first epidemic wave (see Figure 2 in the manuscript), up to the start of ephemeral grouping, and the following basic spreading mechanism on top of which ephemeral groups are created. We include this here for completeness and to ease the understanding.
- Pseudocode for ephemeral groups creation.

Model states, transition diagram, and execution pseudocode

Figure S2 shows model states and transitions. Several simplifying assumptions are made. Propagation is carried out as simple contagion, i.e., purely probabilistic state change when a susceptible agent has a direct link to an agent in an infected state. The model represents a variation of the conventional SIR, with the Infected state decomposed in subclasses: II (Incubating Infected), MI (Mild Infected), AI (Acute Infected), C (Contained). These states differ in the probability of contagion (nominal rate for AI, reduced for II and MI, zero for C) and the time spent by agents in the state (short for II and AI, longer for MI and C)[3,4]. These subclasses could, more generally, be considered as different subpopulations of an heterogeneous class of agents with spreading ability but different degrees of effectiveness and duration. The population of agents is constant, at start up all agents have initial state S (Susceptible), with the exception of the seed agents having state II (Incubating Infected), and state R (Recovered) is final. The list of parameter settings is showed in Table S2.

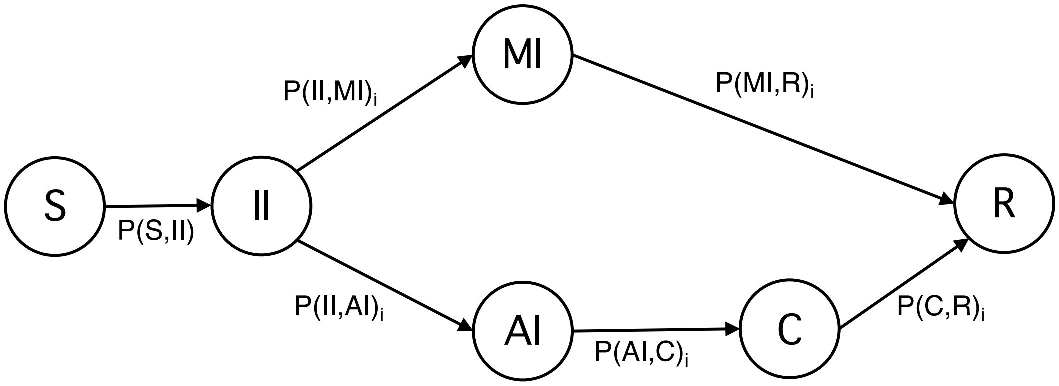

Labels on links represent corresponding transition probabilities from one state to the another.

Figure S2. Model states and transition diagram.

These model assumptions represent a closed system able to produce a SIR-type dynamic (growth-peak-decline), with at most small local stochastic fluctuations, and a final stable state reached in finite time with the complete expiration of the epidemic (zero Infected agents). No oscillatory behavior (e.g., epidemic relapses, multiple epidemic waves, waves with multiple peaks) or stationary states (endemic state) are possible. Among the many aspects that are not considered, some of the most relevant are: a variable population with influx and outflux, a temporal decay of the immunity, the possibility of exogenous events, a finer definition of agent states and transitions. However, for all the improved descriptive ability that an enriched model

may provide, the increased complexity would have complicated the study aimed at introducing a variant of a SIR-type dynamic with ephemeral groups as basic mesoscopic constructs. We believe that only in a second phase of the research, an extended model should be employed by considering a richer set of characteristics. For the purpose of this study, it is also not relevant to specifically consider the different infectious states (II, MI, AI, C). What matters is that overall the basic epidemic dynamic exhibits the characteristics of a SIR-type model, therefore, with no loss of generality, in this work we consider agents in states II, MI, AI, C as aggregated in the generic class I (Infected).

The execution of the original epidemic network model, representing here the dynamic on the static contact network, is described in Algorithm 1. Each iteration represents a time step in simulation time. At every time step, each agent is selected in random order and, if in state S, its state is checked with respect to peers, or if in other states, according to time periods specific of states II, MI, AI, and C.

Ephemeral groups creation pseudocode

The execution of the ephemeral groups model is described in Algorithm 2 and Algorithm 3. Iterations starts when a given condition on the epidemic dynamic is met, corresponding to a certain time step t_G. In our case, we use a condition on the number of agents in class C during the declining phase of the first wave. The rationale for using the class C is discussed in the previous work[2] and it was meant to set a condition on an observable variable and by convention assume it as the start of reopening. For simplicity, we have reused the mechanism in this work too, but a corresponding condition on the aggregate class I would have equally served the same goal. In this work, the rational is that, at a conventional level of infected agents during the declining phase, ephemeral groups start appearing. Changing the level at which ephemeral groups start changes the initial conditions of the grouping process (i.e., number agents in state S, I, R at t_G). In this work, we choose empirically the condition for starting ephemeral groups as one low enough to let us test a large variety of groups configurations, but not so low to make valid trials the exception with respect to invalid ones. In Table S2, the starting conditions of ephemeral groups are showed as average number of Susceptible, Infected and Recovered agents with 95% CI.

Table S2. Base epidemic and ephemeral groups simulation settings.

**Epidemic model**	
**Parameter**	**Values**
Network size	10000
Seed agents	5
Time steps (max)	300
Probability of transmission	AI state: 0.03<br>II & MI states: 0.015<br>C state: 0.0
Length of time in a state	II state: t=[2,14], mean=8<br>MI state: t=[2,7], mean=4.5<br>AI state: t=[2,7], mean=3<br>C state: t=[14,30], mean=22<br>S & R states: undefined
Proportion AI:MI	10:90
**Ephemeral Groups model**	
**Starting conditions at t_G**	**Values** (mean [95% CI])
Infected agents	140 [142.25,137.75]
Susceptible agents	7087 [7122.24,7051.76]
Recovered agents	2773 [2806.25,2739.85]

Algorithm 1 Epidemic network model execution

Require: Adjacency matrix $(A_{i,j})$, random seeds, parameters initialization
for t in $MaxTimesteps$ **do**
for i in $A_{i,j}$ **do**
At each time step t, for all agents in $A_{i,j}$, run the model according to the current agent's state
Case S:
if $Node_i$ in state S **then**
for j in $A_{i,j} = 1$ **do**
if $Node_j$ in state AI **then**
Change state to II with probability P(S,AI)
end if
if $Node_j$ in state MI **then**
Change state to II with probability P(S,MI)
end if
if $Node_j$ in state II **then**
Change state to II with probability P(S,II)
end if
end for
end if
Case II:
if $Node_i$ in state II **then**
Remain in II for $T_{II}(i) = gamma(\alpha_1, mean_1/\alpha_1)$ steps
if $rand[0,1)] < MI/(MI + AI)$ **then**
Change state to MI
else
Change state to AI
end if
end if
Case MI:
if $Node_i$ in state MI **then**
Remain in MI for $T_{MI}(i) = norm(T_{MI})$ steps
When $T_{MI}(i)$ expires:
if $rand[0,1)] < P(MI, C)$ **then**
Change state to C
else
Change state to R
end if
end if
Case AI:
if $Node_i$ in state AI **then**
Remain in AI for $T_{AI}(i) = gamma(\alpha_2, mean_2/\alpha_2)$ steps
When $T_{MI}(i)$ expires, change state to C
end if
Case C:
if $Node_i$ in state C **then**
if $Node_i(t-1)$ changed state from MI **then**
Remain in C for $T_{C|MI}(i) = norm(T_{C|MI})$ steps
else if $Node_i(t-1)$ changed state from AI **then**
Remain in C for $T_{C|AI}(i) = norm(T_{C|AI})$ steps
end if
end if
Case R:
if $Node_i$ in state R **then**
Remain in R
end if
end for
end for

Algorithm 2 Ephemeral groups model execution.

Require: Ephemeral groups starting conditions matched at time step t_G of the epidemic network model execution (Algorithm 1), in the declining phase of the SIR-type dynamic
Require: Adjacency matrix $(A_{i,j})$
Require: y_1: group size, y_2: number of dynamic links per group, y_3: grouping pattern, $\#G$: number of groups

$t_0 \leftarrow t_G$
Grouping pattern is verified from parameter y_3
Possible cases: Consecutive Bounded ($y_3 : n = \bar{n}$), Consecutive Unbounded ($y_3 : f = 1/1$), Periodic Unbounded ($y_3 : f = 1/k$)
Case 1: Consecutive Bounded
if $y_3 : n = \bar{n}$ **then**
    for q in (1, $\bar{n}$) **do**
        CreateGroups($A_{i,j}$, y_1, y_2, y_3, $\#G$)
    end for
end if
Case 2: Consecutive Unbounded
if $y_3 : f = 1/1$ **then**
    for q in (1, $MaxTimesteps - t_G$) **do**
        CreateGroups($A_{i,j}$, y_1, y_2, y_3, $\#G$)
    end for
end if
Case 3: Periodic Unbounded
if $y_3 : f = 1/k$ **then**
    for q in (1, $MaxTimesteps - t_G$) with step k **do**
        CreateGroups($A_{i,j}$, y_1, y_2, y_3, $\#G$)
    end for
end if

Algorithm 3 CreateGroup function.

```
function CreateGroups(A_{i,j}, y_1, y_2, y_3, #G)
for g in #G do
    Randomly select y_1 agents
    for p in y_1 do
        Grp = [append(rand[1,N])]
    end for
    Randomly create y_2 links among selected agents
    for p in y_2 do
        x_1 = rand[Grp]; x_2 = rand[Grp]
        Update the adjacency matrix and possible spread of contagion
        A_{x_1,x_2} = 1
        if x_1 in state S then
            if x_2 in state AI then
                Change state to II with probability P(S,AI)
            end if
            if x_2 in state MI then
                Change state to II with probability P(S,MI)
            end if
            if x_2 in state II then
                Change state to II with probability P(S,II)
            end if
        else if x_2 in state S then
            if x_1 in state AI then
                Change state to II with probability P(S,AI)
            end if
            if x_1 in state MI then
                Change state to II with probability P(S,MI)
            end if
            if x_1 in state II then
                Change state to II with probability P(S,II)
            end if
        end if
    end for
    Remove dynamic links A_{\bar{i},\bar{j}} = 0
end for
```

S3: Null Model analysis

In this section, we present a *null model analysis*, testing the expectation that any patterns in simulation outcomes arise only from random sampling processes (i.e., *null hypothesis*)[5,6]. The *alternative hypothesis* is that patterns in data are not random variations produced by the null hypothesis and therefore assuming the creation of ephemeral groups as relevant for the outcome cannot be rejected. For the analysis, we assume as data the time series with the average number of agents in state Infected, calculated with respect to the simulation trials. Averaging is preceded by the realignment of trials with respect to the flex's time step, which represents the beginning of the second epidemic dynamics. This step has been commented in the manuscript. In the following, we use the expression *Group model* to refer to our original network model based on ephemeral groups.

Random Dynamic Links (RDL) and Constant Random Dynamic Links (CRDL) null models

Two null models have been defined:

- **Random Dynamic Links (RDL)**: Dynamic links are created randomly in each time step of a series of dynamic linking time steps, with agents not organized in groups. A RDL null model is compared to a Group model with the constraint that for each time step of a series of dynamic linking time steps, the total number of random links created in the null model is equal to the sum of links created randomly in all groups of the Group model.
- **Constant Random Dynamic Links (CRDL)**: This is a variation of the RDL null model still having random dynamic links created in the first time step of a series of dynamic linking time steps, but then kept unchanged in the following time steps.

Given the general description of the null models, the competing hypothesis could be stated as follows:

- H_0 : *Null Hypothesis*: The time series of the Infected agents resulting from a Group model could also be explained as resulting from a corresponding RDL or CRDL null model.
- H_1 : *Alternative Hypothesis*: The time series of the Infected agents resulting from a Group model cannot be explained as resulting from one of the null models.

Non-nested model

Before presenting test results, few considerations are needed regarding the rival statistical models we defined for the Group model and for the RDL/CRDL null models in order to perform a significance test. In particular, we defined them as *non-nested models*, meaning that the RDL/CRDL null models are not included in the Group model[7,8].

Formally, the general representation of a non-nested model is a joint probability density function:

$$H_f : f(\mathbf{y}_t|\theta), \theta \in \Theta$$
$$H_g : g(\mathbf{y}_t|\gamma), \gamma \in \Gamma$$

with $f(y_t|\theta)$ and $g(y_t|\gamma)$ two density functions, θ and γ unknown but admissible parameters in parameter spaces Θ and Γ.

In the special case of linear regression models, the one we adopted, the form of the non-nested model is:

$$H_f : \mathbf{y} = \mathbf{X}\alpha + \mathbf{u_f}$$
$$H_g : \mathbf{y} = \mathbf{Z}\beta + \mathbf{u_g}$$

with $\mathbf{X}$ and $\mathbf{Z}$ two vectors of observations on the explanatory variables of models H_g and H_f, u_f and u_g as noise factors with normal distribution, and α and β the unknown regression coefficients[8].

Through a non-nested model, the goal is to evaluate which of the rival models, H_g and H_f, can be considered the best predictor, if any, of the unknown joint probability density function, $f_0(y)$. Therefore, in its general form, the alternatives in a non-nested model simply represent different theories, none of which necessarily has the characteristics of a null model. This is also the reason for the different notation, H_g and H_f instead of H_0 and H_1.

However, since in our case we do test our Group model with respect to the RDL/CRDL null models, for conformity we maintain the traditional H_0 and H_1 notation.

Explanatory variables and linear regression models

Our aim is to test the alternative hypothesis with respect to data represented by the time series with the average number of agents in state Infected. The logic we followed in the definition of the models consider that the algorithm for dynamic link creation is what differs between the hypothesis. Therefore explanatory variables should be defined to reflect the effect of those

different algorithms. By design, the number of dynamic links per time step is not an explanatory variable, because for the alternative models that number is the same. The number and frequency of time steps with dynamic links is defined by the specific grouping pattern (e.g., limited or unlimited consecutive time steps, periodic with a certain frequency). However, there is a key difference between the Group model and the RDL/CRDL null models in the number of dynamic links that *could propagate a contagion*, which are only those connecting a Susceptible agent on one end and an Infected agent at the other end. For simplicity, we refer to these links as *Active links*. Here is the main observation of our hypothesis testing:

The number of Active links, among the total number of dynamic links created in a time step, depends on the different dynamic link creation algorithms of Group model and RDL/CRDL null models, therefore it can be used as explanatory variable for the non-nested models.

Consequently, the linear regression models could be specified as:

**Null Model**	$H_0 : Infected \sim ActiveLinks\_RDLnull$	$H_0 : Infected \sim ActiveLinks\_CRDLnull$
**Group Model**	$H_1 : Infected \sim ActiveLinks\_AllGroups$	$H_1 : Infected \sim ActiveLinks\_AllGroups$

with *Infected* the time series of Infected agents produced by the Group model, and explanatory variables *ActiveLinks_RDLnull*, *ActiveLinks_CRDLnull*, and *ActiveLinks_AllGroups* corresponding to the time series of Active links produced in the three models.

Number of Active links

The probability for a random link to be an Active link for each timestep can be estimated from model simulations. Statistics needed for the evaluation are: *rate of Infected agents* $P_t(Infected)$ and *rate of Susceptible agents* $P_t(Susceptible)$ per time step. From these, the probability $P_t(ActiveLink)$ that, at a certain time step, a dynamic link is an Active link is:

$$P_t(\text{ActiveLink}) = 2 * P_t(\text{Infected}) * P_t(\text{Susceptible})$$

Next, we have estimated the number of *unique links* created per time step. This number is different from the total number of dynamic links created per time step, because dynamic links can be repeated.

The number of unique links, resulting from the random selection with repetition of k links over n possible links, can be evaluated by:

$$UniqueLinks = n\left(1-\left(1-\frac{1}{n}\right)^k\right)$$

which results, for the different models:

**Group Model**	$UniqueLinks\_singlegroup = a\left(1-\left(1-\frac{1}{a}\right)^{y_2}\right)$	and $a = \frac{y_1(y_1-1)}{2}$
**RDL/CRDL Models**	$UniqueLinks\_global = b\left(1-\left(1-\frac{1}{b}\right)^{\#G*y_2}\right)$	and $b = \frac{N(N-1)}{2}$

with, for the Group model, a group of size y_1 and number of dynamic links per time step y_2, while for null models, $\#G * y_2$ represents the total random links created over the entire population N. The following important property holds:

$$UniqueLinks\_global \neq \#G * UniqueLinks\_singlegroup$$

Finally, the *number of Active links* at each time step can be calculated as:

**Group Model**	$ActiveLinks\_AllGroups_t = \#G * UniqueLinks\_singlegroup * P_t(\text{ActiveLinks}\mid\text{Group model})$
**RDL Model**	$ActiveLinks\_RDLnull_t = UniqueLinks\_global * P_t(\text{ActiveLinks}\mid\text{RDL model})$
**CRDL Model**	$ActiveLinks\_RDLnull_t = UniqueLinks\_global * P_t(\text{ActiveLinks}\mid\text{CRDL model})$

with the notation $P_t(\text{ActiveLinks}\mid\text{...model})$ indicating the probability that a random link is an Active link at time step t given Group, RDL, or CRDL model.

Hypothesis testing

With the definition of non-nested model and the three time series of Active links as explanatory variables, resulting from trials of Group model and RDL/CRDL null models simulations, we have been able to test the hypothesis.

As statistical testing methodology, we selected the *Encompassing test* being well-suited for non-nested models[7,9,10].

This test is available as a convenient R function[11] that takes two non-nested linear regression models as input, generically called *Model 1* and *Model 2*, and a data frame containing the time series. Operationally, it defines a new model called *Model E* as the one composed by the explanatory variables of Model 1 and Model 2, so that both original models are nested in the new encompassing model. Then it proceeds to test Model 1 Vs. Model E and Model 2 Vs. Model E. The method used is Wald test for nested models, similar to ANOVA test[12].

The output produces the F statistics of the two tests with the corresponding significance level and degrees of freedom. Checking a F distribution table at a certain significance level (alpha level), first it could be verified if there is a statistically significant difference between the mean scores of Model 1 or Model 2 with respect to the encompassing Model E. If a significant difference is confirmed, then the F statistics obtained by Model 1 and Model 2 could be compared and that with the higher value has the better explanatory power. For example, if Model 1 corresponds to the Group model and Model 2 to the RDL null model, we will have:

**Group Model**	Model 1 : $Infected \sim ActiveLinks\_AllGroups$
**Null Model**	Model 2 : $Infected \sim ActiveLinks\_RDLnull$
**Encompassing Model**	Model E : $Infected \sim ActiveLinks\_AllGroups + ActiveLinks\_RDLnull$

The outcome will have a form similar to:

	Res.Df	Df	F	Pr(>F)
M1 vs. ME	160	-1	309.60	< 2.2e-16 ***
M2 vs. ME	160	-1	189.38	< 2.2e-16 ***

Signif. codes: 0 '***' 0.001 '**' 0.01 '*' 0.05 '.' 0.1 ' ' 1

with *Res.Df* and *Df* respectively the residual and the difference of regression degrees of freedom, *F* the F value, and *Pr(>F)* the significance level. In the example, both models show a significant difference with respect to the encompassing model and Model 1 has a better explanatory power than Model 2. In this case, we can reject the RDL null model.

Results and discussion

Results of hypothesis testing are presented in Table S3 for a sample of the 160 configurations simulated in this work. In particular, for each scenarios defined by $\#G = (10, 100, 1000)$ and each series of simulations with same group size and number of dynamic links per group, (y_1, y_2), we selected for testing the configuration that first reached the peak of Infected. Configurations that plateaued reached the peak in a limited number of time steps (e.g. $y_3 : n = 12$ or $y_3 : n = 16$), others reached the peak with the continuous unlimited pattern ($y_3 : f = 1/1$) in a number of time steps greater of 16. The scenario with $\#G = 1$ is not considered because only a single group of agents is created with random links, therefore it is equivalent to random models. In Table S3, the color code should give an immediate feeling of the test results. A red square means that the null model cannot be rejected; a blue square that the Group model has a better statistical significance. When the F statistics were close we signal it adding "weak".

Results from Table S3 look interesting, we believe, because they show a pattern representing a change of significance of the Group model with respect to random models following the increase of the group size. For the smallest groups there is no statistical difference between the null models and the Groups model; basically small ephemeral groups are effective just because they create new temporal connections, being the group structure unimportant. Then, by increasing the group size, the difference progressively emerges, first weakly, then more evident. For mid-sized to large groups, the fact they have a group structure (even non persistent or temporary, as in our cases) becomes relevant.

This evidence, may have interesting logical explanations with respect to the group behavior, not just between very small and larger groups, but also considering a dynamics of groups, for example starting as small ephemeral groups and progressively forming larger social structures. A conclusion could be that the Null Model analysis points to a relevance of mesoscopic constructs that varies with changes in social organization, groups nature, formation, and aggregation, and that can be included and accounted for in agent-group network models.

The two random models gave same testing results, with respect to the Group model. We suppose that this is due to the fact that Active links are in any case in small numbers and sparse with respect to the whole network, and new Infected agents directly created by Active links are a large minority with respect to the total (similarly to seed nodes with respect to the Infected produced by the epidemic). Therefore, maintaining the same dynamic links, as in the CRDL null model, or recreating anew as

in the RDL null model does not make much difference, in our context. Anyhow, this does not mean that it could not be relevant under different conditions and models or that a similar variant applied to the Group model (i.e., creating persistent groups that maintain constant their members for more timesteps) is not worth studying. We think they could produce interesting results and be the subject of future works.

Table S3. Hypothesis testing: Group model Vs. RDL null model and Group model Vs. CRDL null model. For each test, the alternative with higher F value is showed in bold. Colored boxes help to interpret testing results: **Red** when the Null model has better explanatory power, **Blue** when the Group model has. The notation "weak" is qualitative and indicates when F values between Group and Null models are close. **Parameters**: $\#G$ is the number of groups created per time step, y_1 the group size, y_2 the number of random links per group and per time step, and y_3 the grouping pattern (i.e., a finite number of time steps n or the consecutive unlimited case $f = 1/1$).

**Parameters**	**Model 1: Groups - Model 2: RDL null**		**Model 1: Groups - Model 2: CRDL null**	
**#G=1000**	Model 1: Infected ~ActiveLinks_AllGroups<br>Model 2: Infected ~ActiveLinks_RDLnull<br>Model E: Infected ~ActiveLinks_AllGroups + ActiveLinks_RDLnull<br>—<br>Test - Residual Df - Regression Df - F - Pr(>F)<br>Signif. codes: 0 '***' 0.001 '**' 0.01 '*' 0.05 '.' 0.1 ' ' 1		Model 1: Infected ~ActiveLinks_AllGroups<br>Model 2: Infected ~ActiveLinks_CRDLnull<br>Model E: Infected ~ActiveLinks_AllGroups + ActiveLinks_CRDLnull<br>—<br>Test - Residual Df - Regression Df - F - Pr(>F)<br>Signif. codes: 0 '***' 0.001 '**' 0.01 '*' 0.05 '.' 0.1 ' ' 1"	
y1= 20; y2=100; y3: f=1/1	M1 vs. ME 170 -1 460.66 <2.2e-16 ***<br>**M2 vs. ME 170 -1 776.54 <2.2e-16 *****	(red)	M1 vs. ME 170 -1 285.07 <2.2e-16 ***<br>**M2 vs. ME 170 -1 449.61 <2.2e-16 *****	(red)
y1= 20; y2=200; y3: n=16	M1 vs. ME 167 -1 9.0464 0.0030394 **<br>**M2 vs. ME 167 -1 13.0051 0.0004099 *****	(red) weak	M1 vs. ME 167 -1 16.193 8.657e-05 ***<br>**M2 vs. ME 167 -1 21.223 8.075e-06 *****	(red) weak
y1= 50; y2=250; y3: n=12	**M1 vs. ME 169 -1 5.3465 0.02197 ***<br>M2 vs. ME 169 -1 4.8956 0.02827 *	(blue) weak	**M1 vs. ME 169 -1 7.9032 0.005518 ****<br>M2 vs. ME 169 -1 7.1492 0.008236 **	(blue) weak
y1= 50; y2=500; y3: f=1/1	**M1 vs. ME 167 -1 13.6463 0.0002987 *****<br>M2 vs. ME 167 -1 9.8225 0.0020368 **	(blue) weak	**M1 vs. ME 167 -1 5.5260 0.01990 ***<br>M2 vs. ME 167 -1 3.9671 0.04803 *	(blue) weak
**#G=100**				
y1= 50; y2=500; y3: f=1/1	**M1 vs. ME 160 -1 3500.6 <2.2e-16 *****<br>M2 vs. ME 160 -1 1161.3 <2.2e-16 ***	(blue)	**M1 vs. ME 160 -1 309.60 <2.2e-16 *****<br>M2 vs. ME 160 -1 189.38 <2.2e-16 ***	(blue)
y1= 50; y2=1000; y3: f=1/1	**M1 vs. ME 168 -1 8.5678 0.003896 ****<br>M2 vs. ME 168 -1 0.7394 0.391064	(blue)	**M1 vs. ME 168 -1 178.111 <2.2e-16 *****<br>M2 vs. ME 168 -1 78.296 1.19e-15 ***	(blue)
y1= 50; y2=2000; y3: n=16	**M1 vs. ME 167 -1 37.658 5.924e-09 *****<br>M2 vs. ME 167 -1 13.593 0.0003067 ***	(blue)	**M1 vs. ME 167 -1 37.417 6.554e-09 *****<br>M2 vs. ME 167 -1 14.582 0.0001888 ***	(blue)
y1= 100; y2=1000; y3: f=1/1	**M1 vs. ME 169 -1 312.76 <2.2e-16 *****<br>M2 vs. ME 169 -1 154.90 2.2e-16 ***	(blue)	**M1 vs. ME 169 -1 697.57 <2.2e-16 *****<br>M2 vs. ME 169 -1 265.07 2.2e-16 ***	(blue)
y1= 100; y2=2000; y3: n=16	**M1 vs. ME 170 -1 45.230 2.554e-10 *****<br>M2 vs. ME 170 -1 23.151 3.286e-06 ***	(blue)	**M1 vs. ME 170 -1 45.952 1.910e-10 *****<br>M2 vs. ME 170 -1 25.026 1.401e-06 ***	(blue)
y1= 200; y2=2000; y3: n=16	**M1 vs. ME 169 -1 38.319 4.405e-09 *****<br>M2 vs. ME 169 -1 30.544 1.216e-07 ***	(blue)	**M1 vs. ME 169 -1 38.646 3.841e-09 *****<br>M2 vs. ME 169 -1 31.984 6.506e-08 ***	(blue)
**#G=10**				
y1= 100; y2=6000; y3: f=1/1	**M1 vs. ME 167 -1 4517.34 <2.2e-16 *****<br>M2 vs. ME 167 -1 373.77 <2.2e-16 ***	(blue)	**M1 vs. ME 167 -1 2558.29 <2.2e-16 *****<br>M2 vs. ME 167 -1 730.65 <2.2e-16 ***	(blue)
y1= 200; y2=8000; y3: f=1/1	**M1 vs. ME 173 -1 2264.140 <2.2e-16 *****<br>M2 vs. ME 173 -1 36.645 8.563e-09 ***	(blue)	**M1 vs. ME 173 -1 7443.28 <2.2e-16 *****<br>M2 vs. ME 173 -1 382.17 <2.2e-16 ***	(blue)
y1= 500; y2=20000; y3: n=16	**M1 vs. ME 169 -1 60.707 6.407e-13 *****<br>M2 vs. ME 169 -1 14.042 0.000245 ***	(blue)	**M1 vs. ME 169 -1 80.234 5.907e-16 *****<br>M2 vs. ME 169 -1 11.239 0.0009884 ***	(blue)

S4: Non Overlapping Group model

Another model analysis has been conducted in order to evaluate the statistical difference between the Group model and a variant with the additional constraint of creating only *disjoint groups* in the same time step. We refer to this case as the *Non Overlapping Group model*. The aim of the analysis and the way we proceed are similar to what presented in the previous Null Model analysis section.

The main difference is that, while we still have non-nested linear regression models with explanatory variables represented by the Active links produced in the two cases, differently from the Null Model analysis, now none of the alternatives has the characteristics of a null model. Rather, they are models based on alternative algorithms (i.e., random choice with repetition Vs. random choice without repetition), representing different theories.

Operationally, this requires testing models reciprocally. Therefore, two set of hypothesis are tested.

Case A:

- $H^A_{Overlap}$: The time series of Infected agents produced by the Group model (*Infected_Overlapping*) cannot be explained as resulting from the Non Overlapping Group model.
- $H^A_{NonOverlap}$: The *Infected_Overlapping* time series can be explained as resulting from the Non Overlapping Group model.

Case B:

- $H^B_{NonOverlap}$: The time series of Infected agents produced by the Non Overlapping Group model (*Infected_NonOverlapping*) cannot be explained as resulting from the Group model.
- $H^B_{Overlap}$: The *Infected_NonOverlapping* time series can be explained as resulting from the Group model.

Correspondingly, the linear regression models become:

Case A:

$$H^A_{Overlap} : Infected\_Overlapping \sim ActiveLinks\_AllGroups$$
$$H^A_{NonOverlap} : Infected\_Overlapping \sim ActiveLinks\_NonOverlapping$$

Case B:

$$H^B_{NonOverlap} : Infected\_NonOverlapping \sim ActiveLinks\_NonOverlapping$$
$$H^B_{Overlap} : Infected\_NonOverlapping \sim ActiveLinks\_AllGroups$$

Results and discussion

Results of hypothesis testing are presented in Table S4 for a sample of four configurations with different characteristics. In general, they tend to reject the hypothesis that the dynamics produced by the two models are statistically different, meaning that for groups studied in this work, the possible overlapping does not have dominant effects. Some observations are, however, possible to discuss. For instance, the configuration with parameters ($\#G = 1000$, $y_1 = 10$, $y_2 = 50$) shows some interesting properties:

- All agents of the population ($N = 10000$) belong to an ephemeral group ($\#G * y_1 = 10000$) of size 10. Therefore this is the scenario with highest rate of agents involved in the dynamic link mechanism, but highest group fragmentation (further reducing the group size makes the epidemic unlikely to start).
- It is the case for which the Non Overlapping model has the highest explanatory power, meaning that the effect of overlapping groups is not the relevant factor.

If this result is combined with those of Table S3 showing that for small groups both RDL and CRDL null models cannot be rejected, it could be advanced the explanation that the actual effect of small ephemeral groups on the dynamics is to foster the creation of additional dynamic links, having the group structure no particular relevance with respect to both the overall dynamics (this is the observation we made in the Null Model analysis section) and the possible high-order coupling between different groups.

For the other configurations tested, no model shows a clearly better explanatory power, with F statistics very close between models, both for Case 1 and Case 2. This seems to indicate that groups overlaps is not particularly relevant in our current Group model. As for precedent explanations, this outcome is likely dependent to the limited time span of ephemeral groups, whose agents and dynamic links are recreated randomly at each grouping time steps, every time destroying group overlaps. Trying

Table S4. Model analysis: Group model Vs. Non Overlapping Group model. For each test, the alternative with higher F value is showed in bold. Colored boxes help to interpret testing results: **Blue** when Model 1 has better explanatory power, **Blue** when Model 2 has. The notation "weak" is qualitative and indicates when F values between tested models are close. **Parameters**: $\#G$ is the number of groups created per time step, y_1 the group size, y_2 the number of random links per group and per time step, and y_3 the grouping pattern (i.e.,in this sample always corresponding to the consecutive unlimited case $f = 1/1$).

**Parameters**	**Case 1. Model 1: Groups - Model 2: Non Overlapping**	
	Model 1: Infected_Overlapping ∼ Active_Links_AllGroups<br>Model 2: Infected_Overlapping ∼Active_Links_NonOverlapping<br>Model E: Infected_Overlapping ∼Active_Links_AllGroups + Active_Links_NonOverlapping<br>—<br>Residual Df - Regression Df - F value - Pr(>F)<br>Signif. codes: 0 '***' 0.001 '**' 0.01 '*' 0.05 '.' 0.1 ' ' 1	
#G=1000<br>y1= 10; y2=50; y3: f=1/1	M1 vs. ME 170 -1 460.66 <2.2e-16 ***<br>**M2 vs. ME 170 -1 776.54 <2.2e-16 *** **	
#G=100<br>y1= 50; y2=1000; y3: f=1/1	**M1 vs. ME 167 -1 13.6463 0.0002987 *** **<br>M2 vs. ME 167 -1 9.8225 0.0020368 **	weak
#G=10<br>y1= 200; y2=8000; y3: f=1/1	M1 vs. ME 167 -1 9.0464 0.0030394 **<br>**M2 vs. ME 167 -1 13.0051 0.0004099 *** **	weak
#G=10<br>y1= 100; y2=6000; y3: f=1/1	**M1 vs. ME 169 -1 5.3465 0.02197 * **<br>M2 vs. ME 169 -1 4.8956 0.02827 *	weak
	**Case 2. Model 1: Non Overlapping - Model 2: Groups**	
	Model 1: Infected_NonOverlapping ∼Active_Links_NonOverlapping<br>Model 2: Infected_NonOverlapping ∼Active_Links_AllGroups<br>Model E: Infected_NonOverlapping ∼ Active_Links_NonOverlapping + Active_Links_AllGroups<br>—<br>Residual Df - Regression Df - F value - Pr(>F)<br>Signif. codes: 0 '***' 0.001 '**' 0.01 '*' 0.05 '.' 0.1 ' ' 1"	
#G=1000<br>y1= 10; y2=50; y3: f=1/1	**M1 vs. ME 170 -1 449.61 <2.2e-16 *** **<br>M2 vs. ME 170 -1 285.07 <2.2e-16 ***	
#G=100<br>y1= 50; y2=1000; y3: f=1/1	M1 vs. ME 167 -1 3.9671 0.04803 *<br>**M2 vs. ME 167 -1 5.5260 0.01990 * **	weak
#G=10<br>y1= 200; y2=8000; y3: f=1/1	**M1 vs. ME 167 -1 21.223 8.075e-06 *** **<br>M2 vs. ME 167 -1 16.193 8.657e-05 ***	weak
#G=10<br>y1= 100; y2=6000; y3: f=1/1	M1 vs. ME 169 -1 7.1492 0.008236 **<br>**M2 vs. ME 169 -1 7.9032 0.005518 ** **	weak

again to relate to the results of Table S3, other effects may have stronger influence over the dynamics in our case: firstly, the group structure, which testing results of Table S3 suggest to be increasingly relevant with increasing group size, and secondly the effect on agents outside groups but connected through the static contact network, this suggested by results discussed in the manuscript. A possible hypothesis is that these two effects overcome the one produced by groups overlaps.

At any rate, these possible explanations should be considered as educated guesses supported by a few preliminary theoretical results, at best. As a matter of fact, groups overlaps in dynamic network models of epidemics is a feature extremely relevant and still not well understood. Disjoint groups and randomly overlapping groups are two instances of a continuum described by a variable degree of overlapping among groups and temporal patterns of overlapping. As a subject for future research, group overlapping promises interesting challenges and relevant insights, we believe.

References

1. Holme, P. & Kim, B. J. Growing scale-free networks with tunable clustering. *Phys. review E* **65**, 026107 (2002).
2. Cremonini, M. & Maghool, S. The unknown of the pandemic: An agent-based model of final phase risks. *J. Artif. Soc. Soc. Simul.* **23** (2020).
3. Keeling, M. J. & Eames, K. T. Networks and epidemic models. *J. Royal Soc. Interface* **2**, 295–307 (2005).
4. Brauer, F. Compartmental models in epidemiology. In *Mathematical epidemiology*, 19–79 (Springer, 2008).
5. Gotelli, N. J. & Ulrich, W. Statistical challenges in null model analysis. *Oikos* **121**, 171–180 (2012).
6. Veech, J. A. Significance testing in ecological null models. *Theor. Ecol.* **5**, 611–616 (2012).
7. Pesaran, M. H. Non-nested hypotheses. In *Econometrics*, 167–173 (Springer, 1990).
8. Pesaran, M. H. & Weeks, M. Non-nested hypothesis testing: an overview. *A companion to theoretical econometrics* 279–309 (2001).
9. Davidson, R., MacKinnon, J. G. *et al.* Estimation and inference in econometrics. *OUP Catalogue* (1993).
10. Mizon, G. E. & Richard, J.-F. The encompassing principle and its application to testing non-nested hypotheses. *Econom. J. Econom. Soc.* 657–678 (1986).
11. R Documentation. encomptest: Encompassing test for comparing non-nested models (2021).
12. Fahrmeir, L., Kneib, T., Lang, S. & Marx, B. Regression models. In *Regression*, 21–72 (Springer, 2013).

,
